# The Short Form of the Glasgow Composite Measure Pain Scale in Post-operative Analgesia Studies in Dogs: A Scoping Review

**DOI:** 10.3389/fvets.2021.751949

**Published:** 2021-09-30

**Authors:** Barbara Testa, Jacqueline Reid, Marian E. Scott, Pamela J. Murison, Andrew M. Bell

**Affiliations:** ^1^School of Veterinary Medicine, University of Glasgow, Glasgow, United Kingdom; ^2^NewMetrica Research, Glasgow, United Kingdom; ^3^School of Mathematics and Statistics, University of Glasgow, Glasgow, United Kingdom

**Keywords:** pain, post-operative, behavior, CMPS-SF, dog, metrology, translation, welfare

## Abstract

The measurement and treatment of acute pain in animals is essential from a welfare perspective. Valid pain-related outcome measures are also crucial for ensuring reliable and translatable findings in veterinary clinical trials. The short form of the Glasgow Composite Measure Pain Scale (CMPS-SF) is a multi-item behavioral pain assessment tool, developed and validated using a psychometric approach, to measure acute pain in the dog. Here we conduct a scoping review to identify prospective research studies that have used the CMPS-SF. We aim to describe the contexts in which it has been used, verify the correct use of the scale, and examine whether these studies are well**-**designed and adequately powered. We identify 114 eligible studies, indicating widespread use of the scale. We also document a limited number of modifications to the scale and intervention level, which would alter its validity. A variety of methods, with no consensus, were used to analyse data derived from the scale. However, we also find many deficiencies in reporting of experimental design in terms of the observers used, the underlying hypothesis of the research, the statement of primary outcome, and the use of *a priori* sample size calculations. These deficiencies may predispose to both type I and type II statistical errors in the small animal pain literature. We recommend more robust use of the scale and derived data to ensure success of future studies using the tool ensuring reliable and translatable outcomes.

## Introduction

The translational value of natural companion animal models of pain has recently been highlighted (1, 2). Acute pain is common in veterinary practice and valid measurement of this abstract construct is crucially important as a fundamental prerequisite to effective pain management (3, 4). Translational and veterinary clinical research designed to demonstrate the efficacy of analgesic interventions also relies on the use of valid pain outcome measures (5). However, this can be challenging as pain is an unpleasant multi-dimensional experience with sensory and emotional components, which, by its nature, is not directly measurable in animals as they are unable to self-report.

Historically, acute pain in animals has been measured using behavioral observation quantified with simple tools such as the simple descriptive scale (SDS), numerical rating scale (NRS) and the visual analog scale (VAS) (6). However, these tools are associated with a high level of inter-observer variation and their unidimensional nature may not adequately capture complex constructs like pain (6, 7). The Glasgow composite measure pain scale (CMPS) is a multi-item behavioral pain assessment tool, developed using a psychometric approach, to measure acute pain in the dog (8, 9). The short form of the scale (CMPS-SF) was developed for routine clinical use and comprises six behavioral categories with associated descriptors: vocalization, attention to wound, mobility, response to touch, demeanor and posture/activity (10). The CMPS-SF has been validated for the assessment of acute post-operative pain and importantly the score is linked to an intervention level, which guides the requirement for additional analgesia. To retain the validity of the scale, it should be used as it was originally described and validated, thus preserving its integral measurement properties.

As one of the few validated instruments for acute pain measurement in dogs, the CMPS-SF has been adopted widely in research studies investigating the effect of drugs and interventions on perioperative acute pain. Research studies of this type may be complex and challenging to conduct, requiring careful consideration of factors including group sizes, statistical power, control groups, pain measurement instruments, rescue analgesic provision, and data analysis (11, 12). Of particular concern is the finding that many studies of this type may be underpowered to detect a clinically significant difference (13).

Here we conduct a scoping review of the literature to identify prospective research studies that have used the CMPS-SF to measure acute perioperative pain in the dog. The aim of this study was threefold: (i) describe the use of the CMPS-SF in terms of the features of research studies in which it has been employed; (ii) determine if the CMPS-SF has been adopted in an appropriate manner to give valid results; and (iii) establish whether the study design of clinical trials employing the CMPS-SF is such that these studies are well-designed and adequately powered.

## Methods

### Literature Search

A systematic search of PubMed, CAB abstracts, Web of Science and Google Scholar for papers published between 2007 and 2019 (inclusive) was performed (see Supplementary Table 1). Searches were carried out on each platform using combinations of the following key words (and derivatives): dogs (dog, dogs), the Glasgow Composite Measure Pain Scale—short form (GCMPS-SF, GCMPS, CMPS, CMPS-SF, Glasgow Composite Measure Pain Scale, GCMPS short form, CMPS short form, GCPS), postoperative (post operative, post-operative, postoperative) and pain. We also used the citing articles search feature in Google Scholar and Web of Science to identify any articles citing the original paper describing the development of the CMPS-SF (10).

### Inclusion Criteria

Publications were included if they met the following criteria: (i) use of the Glasgow CMPS-SF to assess pain; (ii) investigating acute post-operative pain; (iii) prospective design; (iv) use of the English language; (v) published in a peer-reviewed journal; (vi) conducted in dogs, and (vii) available in full to the authors. Only English language studies were included because validated translations of the CMPS-SF only recently became widely available (14). Foreign language versions of psychometrically developed scales may not be valid and any assessment of validity must take into account the cultural and linguistic aspects of the target language (14). We felt that the potential inclusion of foreign language versions of the scale would make the interpretation of any results difficult as these would not be comparable without validation.

### Data Extraction and Appraisal

Data extraction and coding was performed by one reviewer (BT) with the coding for each article independently reviewed (AB). Any discrepancies or queries were resolved by discussion and consensus. Before performing the review, a data extraction form was developed to extract information from the studies to fulfill the aims of our investigation and the sections were as described below. All data were derived from the manuscripts themselves or noted as not specified if details of a variable were not given. Authors were not contacted to gather further details.

### Variables Describing the Publications

The year and journal were recorded from the website of the publisher. The country of origin of the research was defined as that of the first author's institution. Pain inducing procedures were classified as soft tissue, neurological or orthopedic surgeries. We also recorded whether cases enrolled in a given study underwent the same single surgical procedure, or whether multiple different procedures were used. Any intervention(s) used in the studies was coded into the classes: analgesic drugs, surgical techniques, regional anesthesia techniques or alternative therapies. The “regional anesthesia techniques” category was used for studies which compared regional anesthesia techniques exclusively to each other.

Any other metrology instruments used for the measurement of pain or nociception alongside the CMPS-SF were recorded. Finally, we assessed whether the CMPS-SF was intended as a primary outcome measure in the study. This was determined to be the case if pain assessment was a major aim specified in the title or if a stated hypothesis or aim involved pain measurement.

### Variables Describing the Use of the CMPS-SF and Measured Data

We determined whether any modifications to the scale had been made. Section B of the scale (locomotion) may be omitted if the animal requires assistance to ambulate and therefore this was not counted as a modification. As an analgesic intervention threshold for the scale has been derived (greater than or equal to a score of 6/24 or 5/20 if section B is omitted), we recorded whether the appropriate intervention level had been used, or if this had been modified. We also recorded details of the number, type and experience of those using the instrument.

The trial design for each study was first classed as either observational, i.e., containing a single group where all animals were treated the same, or controlled, where comparisons were made between two or more groups. We then divided the controlled studies into groups based on their stated hypothesis. Those trials where multiple groups were compared with the aim of disproving the null hypothesis were termed superiority trials, in contrast to those stating they were specifically designed to evaluate either equivalence or non-inferiority. We recorded whether any transformations were applied to CMPS-SF scores prior to statistical testing. We also noted how authors approached the scores arising from animals after any provision of rescue analgesia; specifically, we asked whether these scores were excluded from further statistical analysis and whether a last observation carried forward (LOCF) methodology was used. For controlled trials, the statistical techniques used to compare CMPS-SF scores between groups were classified into the following broad classes, each class potentially encompassing a number of different specific statistical techniques: (i) parametric testing; (ii) non-parametric testing; and (iii) categorical comparisons of GCMP-SF scores after grouping into classes. For non-inferiority/equivalence trials a fourth group was required to allow for those studies using a confidence interval-based approach to non-inferiority testing. When scores from the CMPS-SF were used to guide rescue analgesic provision, we recorded the statistical techniques used to compare rescue analgesic use and whether these involved comparing the proportions of animals rescued between groups or the mean number/dose of rescue analgesics required. We also noted any use of survival analysis statistics to compare the time to rescue between groups.

### Variables Describing the Study Designs

We recorded whether each study was conducted across single or multiple centers. When client owned dogs were used as subjects, we termed these publications clinical studies. Where client owned dogs were not used, we used the term experimental study. Among the controlled studies, we recorded whether the authors clearly stated if the trial was randomized and blinded. We did not however record any further details of these parameters such as methods of blinding or randomization. Controlled studies were also classified by the type of control group used, i.e., the group to which the animals receiving the intervention are compared. In studies with a positive control each group received an analgesic which was assumed to provide the same degree of analgesia, e.g., one non-steroidal anti-inflammatory vs. another. We described studies as negatively controlled if no effective analgesia was present at the time of pain scoring. This may have been due to placebo administration, or in some cases where a short acting analgesic was given at premedication (e.g., pethidine or fentanyl). However, this dichotomous scheme did not satisfactorily classify some publications and hence a third descriptor was used, pseudo-negative. In these studies, all groups had some form of analgesia present at the majority of timepoints of pain scoring. However, in one group, the analgesic or combination of analgesics will be potentially less effective. An example of such a study would be where a nerve block is compared to sham but all dogs in the study received an NSAID pre-operatively.

We recorded the number of groups in each study, alongside the mean group size for each study and whether there was a >20% discrepancy in group sizes. While a discrepancy in group sizes is not necessarily problematic (15, 16), the 20% threshold was arbitrarily defined as a level that was considered significant before data collection.

We determined the number of studies that had conducted an *a priori* sample size calculation and, among those, we recorded if the number of cases required was declared and whether sufficient dogs were recruited. In studies where we had determined pain, as measured by the CMPS-SF, to be a primary outcome, we noted whether a sample size calculation was based specifically on pain score data. In order to evaluate the quality of sample size calculations, we used established criteria from the Consolidated Standards of Reporting Trials (CONSORT) guidelines (17). The following elements were required for a study to be categorized as complying with CONSORT sample size guidelines: (i) the clinically important target difference between the groups; (ii) the α (type I) statistical error level; (iii) the statistical power (or the β (type II) statistical error level); (iv) the standard deviation (SD) of the measurements; and v) the source of the standard deviation used in the sample size analysis. We calculated a score out of ten for each sample size calculation based on the information provided. Two points were allocated for appropriate details given for each of the five required elements. With respect to the source of SD values, we allocated a single point to studies using unpublished preliminary data, and two points where a published study was cited as the source.

Finally, we recorded whether a statistically significant difference was found in each of the controlled superiority studies and whether this reflected differences in absolute pain scores, the provision of rescue analgesia, or both.

### Statistical Analysis

All coded variables were recorded in Microsoft Excel (Microsoft, Washington, U.S.). Summary statistics were generated in Jamovi (The Jamovi project, Sydney, Australia). A Mann-Whitney U test was used to compare group sizes between subgroups (non-inferiority vs. superiority and sample size calculation vs. no sample size calculation) with the *p*-value for significance set at <0.05.

## Results

**Figure 1:**
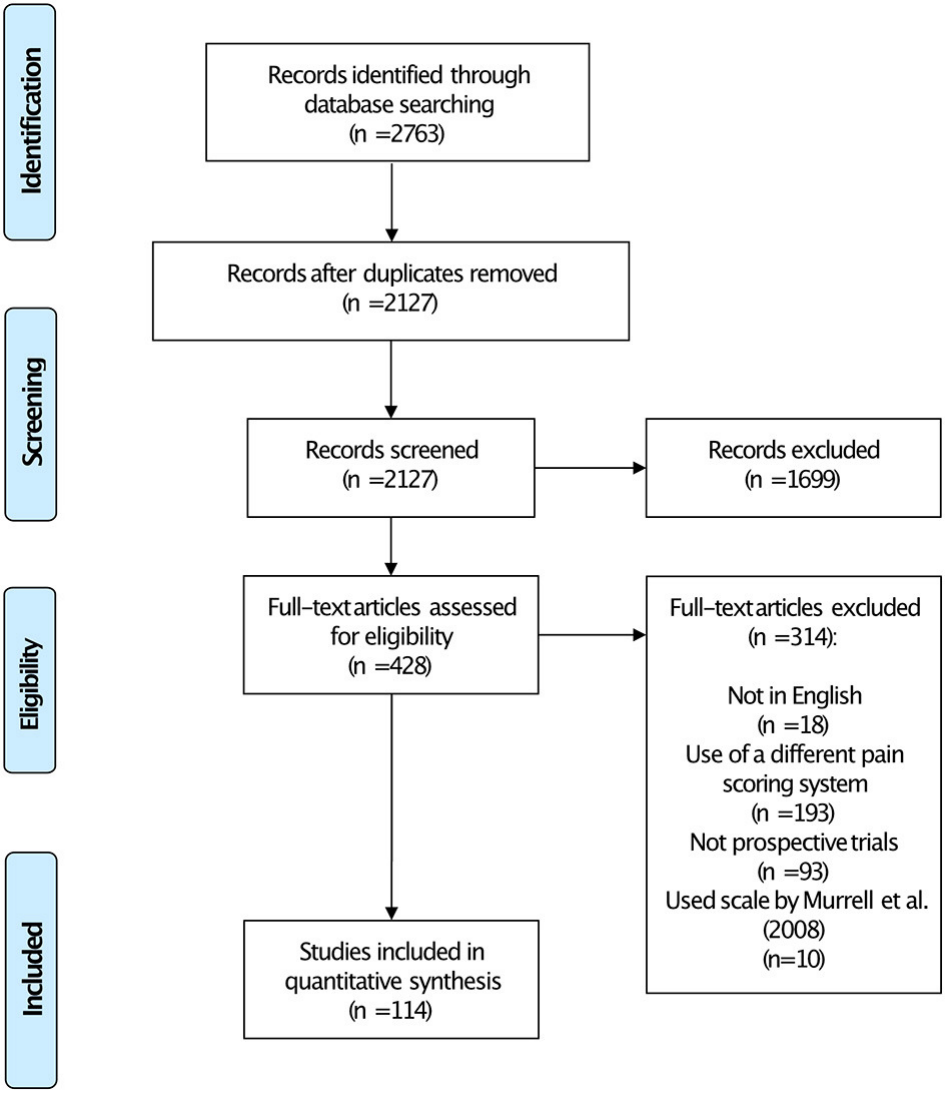
Preferred Reporting Items for Systematic Reviews and Meta-Analyses (PRISMA) flowchart showing the number of studies included in each stage of the review.

We identified 2,763 records through the database search. Following removal of duplicates, screening and full text eligibility assessment, 114 studies were finally included in the scoping review (Figure 1 and Supplementary Table 2).

### Variables Describing the Publications

**Figure 2:**
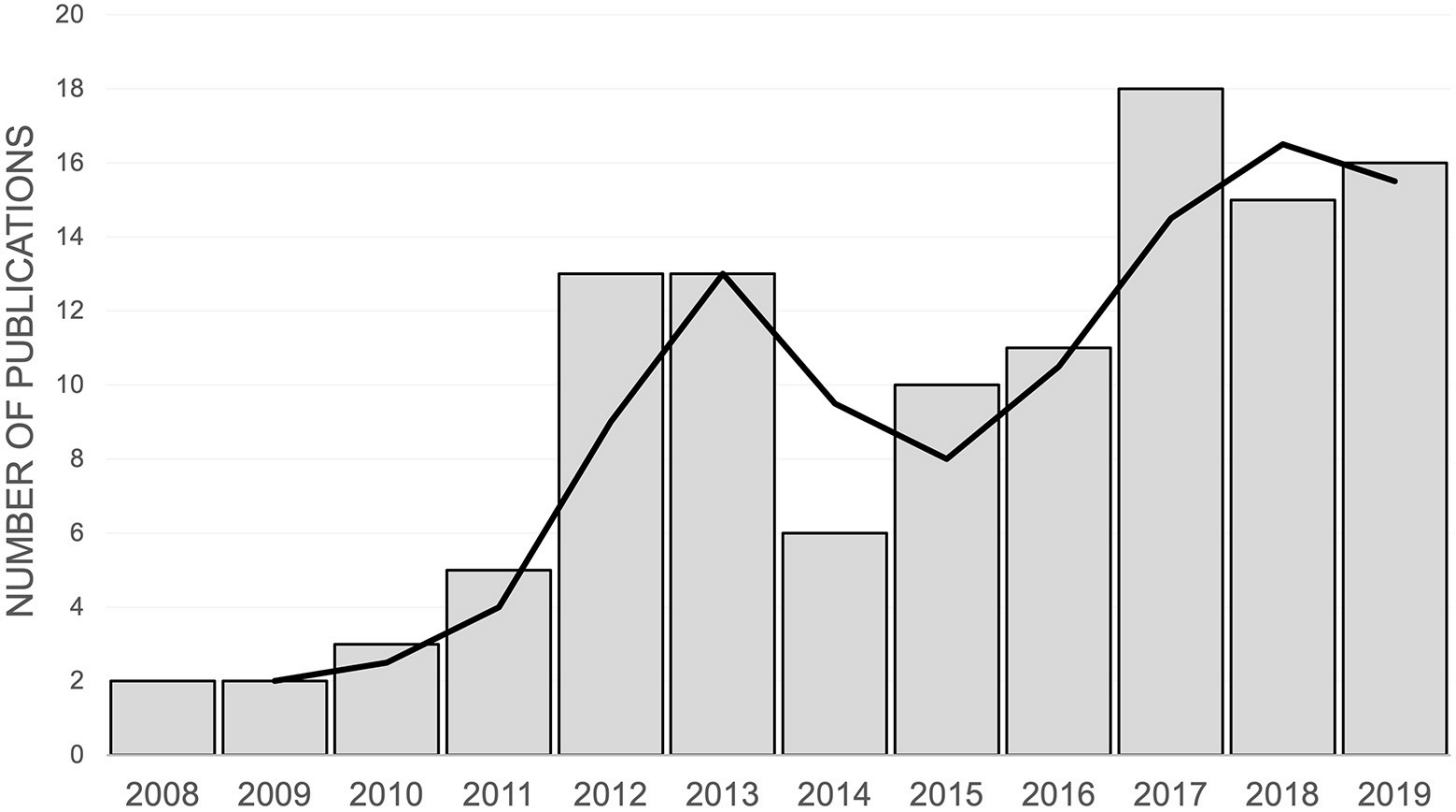
The number of publications using the CMPS-SF by year of publication between 2007 and 2019. The trendline represents a 2-year rolling average of the number of publications.

The numbers of studies employing the CMPS-SF per year are shown in Figure 2. The journals in which the studies were published and the country of origin of the research are described in Table 1. Single soft tissue and orthopedic surgeries accounted for the majority of pain inducing procedures in the eligible studies (Table 1 and Supplementary Table 2). The most common interventions investigated were analgesic drugs (Table 1). In 43% of the studies, another metrology instrument that measured pain or nociception was used alongside the CMPS-SF (Table 1). Furthermore, we established that the CMPS-SF represented a primary outcome measure in 73% of the studies included in this review.

**Table 1:** Variables describing the publications included in the review.

**Variable**	**Category**	***N* =**		**%**
Journal	Veterinary Anesthesia & Analgesia	29		25%
	Journal of the American Animal Hospital Association	11		10%
	Veterinary Surgery	10		9%
	The Veterinary Journal	7		6%
	Journal of Small Animal Practice	7		6%
	BMC Veterinary Research	6		5%
	American Journal of Veterinary Research	5		4%
	Journal of Veterinary Pharmacology and Therapeutics	3		3%
	Journal of Veterinary Internal Medicine	3		3%
	Journal of Veterinary Behavior	3		3%
	Veterinarni Medicina	3		3%
	Journals with fewer than 3 articles (*n* = 21)	27		24%
		114		100%
Country of origin	USA	36		32%
	UK	18		16%
	Italy	12		11%
	Spain	7		6%
	Canada	5		4%
	Ireland	4		4%
	Switzerland	4		4%
	China	3		3%
	Countries with fewer than 3 studies (n=19)	25		22%
		114		100%
Pain Inducing Procedure	Single Soft Tissue Procedure	48		42%
	Single Orthopedic Procedure	20		18%
	Mixed Soft Tissue Procedures	15		13%
	Mixed Procedures	12		11%
	Single Neurological Procedures	11		10%
	Mixed Orthopedic Procedures	8		7%
		114		100%
Analgesic intervention assessed	Analgesic Drugs	79		69%
	Surgical Techniques	15		13%
	Regional Anesthesia Techniques	14		12%
	Alternative Therapies	6		5%
		114		100%
Other “Pain” metrology instruments used	No Yes	65 49		57% 43%
	*VAS*		*28*	
	*Mechanical thresholds*		*21*	
	*Other Composite Pain scale*		*9*	
	*NRS*		*7*	
	*Gait Analysis*		*3*	
	*Electroencepalography*		*2*	
	*Serum biomarkers*		*2*	
		114		100%
Was GCMPS a primary outcome measure?	Yes	83		73%
	No	31		27%
		114		100%

*In the section describing other metrology instruments, the counts of studies in italics are not mutually exclusive*.

*Note: Text in italics denotes subgroups*.

### Variables Describing the Use of the CMPS-SF and Measured Data

The great majority of studies included in this review did not modify the CMPS-SF (Table 2). However, in 7% of the publications some modification was evident. During the course of our review, we found 10 studies which purported to use the CMPS-SF but on closer inspection actually used a modified version of the scale as proposed by Murrell et al. (18). These studies were excluded from our analysis (Figure 1). In most studies investigated, the intervention level for rescue analgesia used was clearly stated as recommended for the scale (Table 2). However, changes to the intervention level were described in around a third of the studies. In some cases, these increases were only by one point (12 of 30 instances), although the mean increase in the intervention level was to 38% of the maximum CMPS-SF score (~9/24, with a range of 7–18). An intervention score in excess of 10/24 was used by 7 papers in this review. We also determined details of the observers who performed scoring in each publication (Table 2).

**Table 2:** Variables from publications in the review describing how the CMPS-SF was used.

**Variable**	**Category**	***N* =**		**%**
Modifications to the scale	No	106		93%
	Yes	8		7%
	*Omit/alter section A*		*5*	
	*Omit section C*		*2*	
	*Combine with physiological data*		*1*	
		114		100%
Intervention level (for non-modified scale)	≥5/20 or ≥6/24	57		54%
	Increased	30		28%
	Decreased	3		3%
	Not specified/Based on other metrology (e.g., VAS)	16		15%
		106		100%
*Observer*				
Number	Single	51		45%
	Not specified	18		16%
	Multiple	45		39%
	*2*		*21*	
	*3*		*3*	
	*4*		*1*	
	*not specified*		*20*	
		114		100%
Pain scoring experience	Experienced/trained	34		30%
	Inexperienced	3		3%
	Not specified	77		68%
		114		100%
Type	Veterinary surgeon	32		28%
	Nurse/technician	5		4%
	Veterinary student	2		2%
	Mixed	4		4%
	Not specified	71		62%
		114		100%

*Note: Text in italics denotes subgroups*.

Of the 114 studies included in the review, 104 (91%) were controlled studies comparing two or more groups. We classed 85 (82%) of these as superiority and 19 (18%) as either equivalence or non-inferiority studies. A variety of statistical approaches were used in the controlled studies to prepare and analyse CMPS-SF data, and these are summarized in Table 3. All trials compared absolute pain scores between groups in some manner, and a smaller proportion (42 of 85 superiority studies and 15 of 19 non-inferiority studies) compared the use of rescue analgesia.

**Table 3:** A summary of handling data from the CMPS-SF and the statistical techniques used.

**Variable**	**Category**	***N* =**	**%**
Data transformed prior to statistical testing?	No/not specified	80	77%
	Transformed to normal (e.g., log transform)	10	10%
	Area under curve	5	5%
	Percentage of possible max	4	4%
	Pooled into classes	4	4%
	Change relative to baseline	1	1%
		104	100%
Data excluded after rescue analgesia?	Yes	32	31%
	No	45	43%
	Not applicable - no rescue required	5	5%
	Both analyses performed	3	3%
	Not specified	19	18%
		104	100%
LOCF Stated as being used	Yes	7	20%
	No	25	80%
		32	100%
Statistics for superiority trials			
Comparing pain scores	Parametric	36	42%
	Non-parametric	33	39%
	Categorical	1	1%
	No formal statistical testing	2	2%
	Not specified	13	15%
		85	100%
Comparing rescue analgesia use	Proportions requiring rescue compared	26	62%
	Means of rescue analgesic administration compared	5	12%
	Both means and proportions compared	4	10%
	Survival analysis (time to rescue) conducted	13^*^	31%^*^
		42	100%
Statistics for non-inferiority trials			
Comparing pain scores	Parametric	6	32%
	Non-parametric	5	26%
	Non-inferiority confidence intervals	2	11%
	Categorical	1	5%
	Not specified	5	26%
		19	100%
Comparing rescue analgesia use	Proportions requiring rescue compared	9	60%
	Means of rescue analgesic administration compared	4	27%
	Survival analysis (time to rescue) conducted	3^*^	19%*
		15	100%

*104 studies are included in this table and the 10 observational studies in the review omitted*.

* *Where numbers of studies using survival analysis are given, these may also be accounted for in the other groupings for comparing rescue analgesia use*.

### Variables Describing the Study Designs and Power

Details of the study designs used in this review are detailed in Table 4. Of the 104 controlled trials, 50 (48%) had conducted an *a priori* sample size calculation for any outcome measure. In 48 of the 50 cases, the total number of dogs required was declared and in 41 of those cases sufficient dogs were recruited. The sample size calculation was performed as per CONSORT guidelines in 12 (24%) of the studies and the median sample size calculation score allocated was 6 (range 2–10). The CMPS-SF represented a primary outcome measure in 36 of the 50 studies with sample size calculations, and yet a sample size calculation related specifically to the CMPS-SF in only 24 (67%) of these. During coding of the studies, we noticed larger group sizes in those with a non-inferiority vs. superiority design (50 ± 69 vs. 21 ± 30 (mean SD), *p* = 0.017) and in those that included a sample size calculation compared to those without (36 ± 53 vs. 18 ± 22, *p* = 0.001).

**Table 4:** Variables describing features of study design in the publications.

**Variable**	**Category**	***N* =**	**%**
Study design			
Center	Single center	101	89%
	Multi center	13	11%
		114	100%
Setting	Clinical	104	91%
	Experimental	10	9%
		114	100%
Cross over design	Yes (all within experimental studies)	2	2%
	No	112	98%
		114	100%
Randomized			
Among controlled studies (*n* = 104)	Yes	104	100%
	No	0	0%
		104	100%
Blinded	Yes	90	87%
	No	14	13%
		104	100%
Control	Positive	60	58%
	Pseudo-negative	31	30%
	Negative	13	13%
		104	100%
Number of groups	Two	75	72%
	Three	20	19%
	4 or greater	9	9%
		104	100%
Dogs per group	Mean ± SD		27 ± 41
	Median (range)		15 (5–251)
>20% size discrepancy?	Yes	7	7%
	No	97	93%
		104	100%

We restricted further analysis of study findings to the 85 controlled studies with a superiority hypothesis. In 38 (45%) of these studies, statistically significant differences were evident, and this occurred between absolute scores (*n* = 21, 55%), guiding of rescue (*n* = 4, 11%), and both measures (*n* = 13, 34%).

## Discussion

In this review, we demonstrate the widespread international use of the CMPS-SF in the canine post-operative analgesia literature. The scale has been applied broadly across investigations into the effect of many different analgesic interventions on pain induced by a variety of surgical interventions. This popularity is perhaps unsurprising given the properties of the scale; namely that it is one of only a few validated tools for the measurement of acute pain in the dog (19–21), and that the scale has a high utility and a defined intervention level (10).

Our results demonstrate a number of noteworthy issues relating to the appropriate use of the scale and the design of the trials in which it has been employed. These considerations have the potential to significantly affect the outcome of studies. Therefore, mitigating against potential shortcomings as described below will be vital to the success of future veterinary clinical research using the CMPS-SF and its translational potential.

### Appropriate Use of the CMPS-SF and Derived Data

The CMPS-SF was developed using a psychometric approach and the validity is dependent on it being used as intended. Modifications to the scale, conducted without revalidation, change the measurement properties and should be avoided. Modifications were found in 7% of the papers in this review, and it is reassuring that this practice is rare. The defined intervention level is also no longer valid if changes are made. We documented a significant number of studies in which the intervention level had been altered. Some of these may simply have been due to poor reporting (stating “greater than” rather than “greater than or equal to”), however many changes were intentional, lacked supporting documentation and therefore were presumably based purely on author opinion. The intervention level was derived during a multi-center clinical study at three separate veterinary hospitals, using animals that had undergone a variety of surgical procedures (10). It is possible that in some other contexts the score may need to be refined to better reflect the needs of a certain population (e.g., feral dog neutering), and novel data would ideally be presented in support of this. It does however seem unlikely that substantial changes in the intervention score (i.e., >10) would be appropriate in any context. Indeed, some of the increases, including an intervention level of 18, detected in this review raise ethical considerations, as animals in severe pain would not receive rescue analgesia.

An aspect of CMPS-SF use that is poorly reported in the literature presented here, and has the capacity to significantly alter results, is the number and the experience of the observer(s) conducting the scoring. By using specific descriptors, the scale is designed to reduce respondent bias and decrease the interobserver variability that has been reported with unidimensional subjective pain scales (6). Among expert observers this would appear to be the case when scoring videos of painful dogs (12), although the use of inexperienced observers is not recommended as agreement may be poor (22).

We detected a lack of consensus regarding the statistical approach to absolute CMPS-SF scores. The statistical test used should reflect the nature of the measurement, and the short form of the CMPS is a non-interval level measure (9). The choice of analysis may also need to be pragmatic to account for complexity of the data, such as repeated measures taken from the same individual. A number of different transformations have been applied to CMPS-SF data prior to statistical testing, predominantly to normalize the data and utilize more powerful parametric statistics. Given the non-interval nature of CMPS-SF data, pooling into classes (representing no pain, mild pain etc.) is a highly appropriate technique, but was only used in a minority of studies, perhaps as cut-off values are likely to be arbitrarily defined. A number of different approaches for dealing with scores arising after animals had received rescue analgesia were also evident, including whether imputation techniques such as LOCF were used. A lack of consensus in this regard also exists in the human acute pain literature (23). A minority of studies in this review sought to evaluate equivalence, however very few of these used the most appropriate statistical approach to this, namely defining a non-inferiority margin and calculating confidence intervals (24).

### The Design of Acute Pain Clinical Trials

We also examined the trials using the instrument in terms of their design and statistical power. Appropriate blinding and randomization are crucial in clinical trials to prevent bias. Significant deficits in reporting have been shown in this respect in the veterinary literature (25, 26). Consistent with this, we noticed during our coding of the data that authors would frequently state the trial was randomized and blinded without giving explicit details. More detailed assessment of these features, e.g., the extent of blinding, is a core part of risk of bias assessments. However, we chose not to conduct these assessments in detail during this review as our investigations centered on the use of pain scoring outcomes rather than establishing (*via* subsequent metanalysis) whether a particular outcome was well-evidenced across a number of studies.

A limited number of trials which used no effective analgesics in the control group (negative controls) were included in this review despite studies of this design often resulting in larger outcome effect sizes. This infrequency likely reflects the possibility of undertreatment of pain in placebo-treated participants and the ethical implications of this which are a significant consideration in veterinary medicine (11) as in human medicine (27).

The number of animals enrolled per group in studies in this review seems relatively low and may be associated with a limited power to detect a significant difference. We observed significantly greater group sizes in non-inferiority trials which may be a reflection of the statistical approach required to demonstrate non-inferiority. We also show that group sizes are larger in studies where an *a priori* sample size calculation is carried out. Major deficits in the power of small animal analgesia studies were identified in literature from over 15 years ago (13). Although our methodology is different, our data would suggest that justification of adequate statistical power is still a significant issue in the small animal pain literature, and this issue is seen more broadly across veterinary clinical trials (26, 28). We also find other deficits in methodologies relating to statistical power. Many studies used pain measurement as a primary outcome, however in some cases a single sample size calculation was conducted for another primary outcome measure, such as anesthetic requirement. This could result in a study underpowered to detect differences in pain scores and a consequent type II statistical error. Additionally, many of the published sample size calculations do not comprise sufficient information to judge their appropriateness. Publication of animal research is often dependent on the inclusion of a sample size calculation in order to satisfy ARRIVE (Animal Research: Reporting of *In Vivo* Experiments) guidance (29). However, our data would suggest this guidance is not being universally applied.

Within pain outcome measures, absolute scores and the requirement for rescue appear to be used interchangeably as measures of efficacy in the canine pain literature. The use of multiple outcome measures to define analgesic success has been promoted recently (30). However, the use of multiple primary outcome measures without a multiple-comparisons adjustment of the threshold for significance may predispose to type I statistical errors (31, 32), even if each component part is underpowered. As our review spans the period during which the CMPS-SF has been in existence, it is conceivable that authors of earlier studies did not have access to preliminary data upon which to base a sample size calculation. However, now that a significant body of CMPS-SF data is available across a number of contexts, this should not be the case. Promoting accessibility of CMPS-SF data will be important to encourage appropriate experimental design using *a priori* sample size calculations in future.

### Limitations

There are number of potential limitations to our findings. Firstly, despite using broad search terms, there is a possibility that we have not included some eligible publications that used the CMPS-SF and did not mention it in a way that was captured by our search. Furthermore, a number of the coded variables (e.g., superiority vs. equivalence, or identification of primary outcome) were coded somewhat subjectively based on the information that was available and this may not have been as originally intended by the primary authors. This reflects deficiencies in reporting evident in some of the included studies and is mirrored more widely in the analgesia literature (29, 33). This is especially important as poor quality of reporting may be associated with finding exaggerated effects (34). A number of solutions to this problem have been proposed, including submission checklists (33, 35). Prior registration of clinical trials is also an essential requirement in human studies, and requires that primary outcome measures, hypotheses, sample size calculations and proposed statistical testing are declared before commencing the trial. Trial registries are in their infancy in veterinary medicine (36), but, based on our findings, are to be recommended to those conducting companion animal pain research.

In conclusion, this review demonstrates widespread use of the CMPS-SF across the canine acute pain literature. For the most part, the scale has been adopted in a valid manner with only a few reported modifications to the scale and the intervention level. However, we document several deficiencies in experimental reporting and design which may predispose to both type I and type II statistical errors.

## Data Availability Statement

The raw data supporting the conclusions of this article will be made available by the authors, without undue reservation.

## Author Contributions

AB and BT conceived and designed the study, extracted data from the included studies, and produced summary data. BT performed the literature search and wrote sections of the manuscript. AB, BT, JR, MS, and PM contributed to discussions classifying studies and interpreting the findings. AB wrote the first draft of the manuscript. All authors contributed to manuscript revision, read, and approved the submitted version.

## Funding

The authors declare that this study received funding from Newmetrica Ltd. for open access fees. The funder itself was not involved in the study design, collection, analysis, interpretation of data, the writing of this article or the decision to submit it for publication.

## Conflict of Interest

JR is a director in NewMetrica Ltd., a company that distributes the Glasgow CMPS-SF for clinical and research use, under license from the University of Glasgow. The remaining authors declare that the research was conducted in the absence of any commercial or financial relationships that could be construed as a potential conflict of interest.

## Publisher's Note

All claims expressed in this article are solely those of the authors and do not necessarily represent those of their affiliated organizations, or those of the publisher, the editors and the reviewers. Any product that may be evaluated in this article, or claim that may be made by its manufacturer, is not guaranteed or endorsed by the publisher.
