# Supplementary material for: The Short Form of the Glasgow Composite Measure Pain Scale in Post-operative Analgesia Studies in Dogs: A Scoping Review
Source: Front Vet Sci. 2021 Sep 30;8:751949. doi: 10.3389/fvets.2021.751949 (PMC8515184; doi:10.3389/fvets.2021.751949)
Supplement: Supplementary file 1 [file Data_Sheet_1.pdf]

Table S1 – Studies Using the CMPS-SF included in the review. TPLO: tibial plateau leveling osteotomy; EHPSS: extra-hepatic portosystemic shunt; C-section: cesarean section.

Reference	Year	Clinical/ Experimental	Observational vs Comparative	Procedure	GCMPS-SF as Primary or Secondary Outcome
Adami et al. (1)	*2016*	Clinical	Controlled	TPLO	Primary
Adami et al. (2)	*2012*	Clinical	Controlled	TPLO	Primary
Aengwanich et al. (3)	*2019*	Clinical	Observational	Castration	Primary
Aghighi et al. (4)	*2012*	Clinical	Controlled	Hemilaminectomy	Primary
Amenegual et al. (5)	*2017*	Clinical	Controlled	Spinal decompressive surgery	Secondary
Andreoni et al (6)	*2009*	Clinical	Observational	Various elective surgeries	Secondary
Aprea et al (7)	*2012*	Clinical	Controlled	Dorsal hemilaminectomy	Primary
Barker et al (8)	*2013*	Clinical	Controlled	Hemilaminectomy	Primary
Barnes et al (9)	*2019*	Clinical	Controlled	TPLO	Primary
Bartel et al (10)	*2016*	Clinical	Controlled	Stifle arthroplasty	Primary
Bellei et al (11)	*2011*	Clinical	Observational	Spinal surgery	Primary
Bendinelli et al (12)	*2018*	Clinical	Controlled	Combined laparoscopic ovariectomy and laparoscopic-assisted gastropexy	Primary
Benitez et al (13)	*2015*	Clinical	Controlled	TPLO	Primary
Benitez et al (14)	*2015*	Clinical	Controlled	TPLO	Secondary
Bienhoff et al (15)	*2011*	Clinical	Controlled	Dental surgery (dental extraction)	Primary
Bienhoff et al (16)	*2012*	Clinical	Controlled	Soft tissue surgery	Primary
Bustamante et al (17)	*2018*	Clinical	Controlled	Ovariohysterectomy	Primary
Caniglia et al (18)	*2012*	Clinical	Controlled	TPLO	Primary

Cerasoli et al (19)	*2017*	Clinical	Controlled	TPLO	Primary
Chiavaccini et al (20)	*2017*	Experimental	Controlled	Thoracic skin incisions	Primary
Dancker et al (21)	*2019*	Clinical	Controlled	EHPSS attenuation	Primary
Davila et al (22)	*2013*	Clinical	Controlled	TPLO	Primary
Fitzpatrick et al (23)	*2010*	Clinical	Controlled	Ovariohysterectomy	Primary
Fizzano et al (24)	*2017*	Experimental	Controlled	Rhinoscopy and nasal biopsies	Secondary
Fransson et al (25)	*2015*	Clinical	Controlled	Laparoscopic Ovariohysterectomy	Primary
Friton et al (26)	*2017*	Clinical	Controlled	Soft tissue surgery	Primary
Friton et al (27)	*2017*	Clinical	Controlled	Soft tissue surgery	Primary
Giudice et al (28)	*2017*	Clinical	Controlled	Hemilaminectomy (acute vertebral disc extrusion)	Primary
Goudie-DeAngelis et al (29)	*2016*	Clinical	Controlled	Ovariohysterectomy	Primary
Groppetti et al (30)	*2019*	Clinical	Observational	C-section	Secondary
Gruet et al (31)	*2011*	Clinical	Controlled	Major orthopaedic surgery	Primary
Gruet et al (32)	*2013*	Clinical	Controlled	Major soft tissue surgery	Primary
Guerrero et al (33)	*2015*	Clinical	Controlled	Ovariohysterectomy	Primary
Guerrero et al (34)	*2016*	Clinical	Controlled	Ovariohysterectomy	Primary
Guillot et al (35)	*2011*	Experimental	Controlled	Bone Marrow Aspirate	Primary
Guimaraes Alves et al (36)	*2014*	Clinical	Controlled	Femoral, tibial, humeral or radial fracture repair	Primary
Gurney et al (37)	*2012*	Clinical	Controlled	Unilateral elbow arthroscopy	Primary
Gutierrez-Bautista et al (38)	*2018*	Clinical	Controlled	Orthopaedic surgery	Primary
Gutierrez-Blanco et al (39)	*2015*	Clinical	Controlled	Ovariohysterectomy	Primary

Hamilton et al (40)	*2014*	Clinical	Controlled	Orchidectomy	Secondary
Heffernan et al (41)	*2018*	Clinical	Controlled	TPLO	Primary
Hettlich et al (42)	*2017*	Clinical	Controlled	Hemilaminectomy	Primary
Hu et al (43)	*2017*	Clinical	Controlled	Ovariohysterectomy	Primary
Hunt et al (44)	*2013*	Clinical	Controlled	Orthopaedic surgery	Primary
Hunt et al (45)	*2013*	Clinical	Controlled	Various surgeries	Primary
Hunt et al (46)	*2014*	Clinical	Controlled	Mixed surgeries	Primary
Huuskonen et al (47)	*2013*	Clinical	Controlled	Castration	Secondary
Kaka et al (48)	*2018*	Clinical	Controlled	Ovariohysterectomy	Primary
Karrasch et al (49)	*2015*	Clinical	Controlled	Cutaneous tumour removal	Primary
Kibar et al (50)	*2019*	Clinical	Controlled	Ovariohysterectomy	Primary
Kim J.H. et al (51)	*2018*	Experimental	Controlled	Arthroscopic surgery (shoulder)	Primary
Kim Y.K. et al (52)	*2012*	Clinical	Controlled	Laparoscopic ovariohysterectomy	Primary
Kondo et al (53)	*2012*	Clinical	Controlled	Soft tissue surgery	Primary
Kongara et al (54)	*2012*	Clinical	Controlled	Ovariohysterectomy	Primary
Kongara et al (55)	*2013*	Clinical	Controlled	Castration	Primary
Kropf et al (56)	*2018*	Clinical	Controlled	Ovariohysterectomy	Secondary
Kropf et al (57)	*2019*	Clinical	Controlled	Ovariohysterectomy or castration	Secondary
Kushnir et al (58)	*2017*	Clinical	Controlled	Castration	Primary
Lambertini et al (59)	*2018*	Clinical	Controlled	Ovariohysterectomy	Primary
Lardone et al (60)	*2017*	Clinical	Controlled	Hip arthroplasty	Primary
Lascelles et al (61)	*2016*	Clinical	Controlled	Lateral retinacular suture procedure, including stifle arthrotomy	Primary
Lewis et al (62)	*2014*	Clinical	Controlled	TPLO	Primary
Li et al (63)	*2017*	Experimental	Controlled	Ovariohysterectomy	Secondary

Linton et al (64)	*2012*	Clinical	Controlled	Soft tissue or orthopaedic surgery	Primary
Little et al (65)	*2016*	Experimental	Observational	Surgical removal of cartilage from the head of the femur	Secondary
Luna et al (66)	*2015*	Clinical	Controlled	Ovariohysterectomy	Primary
Lush et al (67)	*2018*	Clinical	Observational	Castration	Primary
Martinez et al (68)	*2014*	Clinical	Controlled	Mixed surgeries	Primary
Martinez-Taboada et al (69)	*2017*	Clinical	Controlled	Elective surgery of the pelvic limbs or caudal abdomen	Secondary
McCally et al (70)	*2015*	Clinical	Controlled	TPLO	Primary
McKune et al (71)	*2014*	Clinical	Controlled	Ovariohysterectomy	Primary
McMillan et al (72)	*2012*	Clinical	Controlled	Castration	Secondary
Meakin et al (73)	*2016*	Clinical	Controlled	Abdominal surgeries, midline celiotomy	Secondary
Merema et al (74)	*2017*	Clinical	Controlled	Ovariohysterectomy	Primary
Meunier et al (75)	*2019*	Clinical	Controlled	Sterilisation	Primary
Morgaz et al (76)	*2013*	Clinical	Controlled	Ovariohysterectomy	Primary
Morgaz et al (77)	*2014*	Clinical	Controlled	Ovariohysterectomy	Primary
Nour et al (78)	*2013*	Experimental	Controlled	Not specified	Primary
Palomba et al (79)	*2019*	Clinical	Controlled	TPLO	Primary
Pascal et al (80)	*2013*	Clinical	Controlled	Genital surgery	Primary
Pascal et al (81)	*2019*	Clinical	Controlled	Hemilaminectomy	Primary
Peeters et al (82)	*2011*	Clinical	Controlled	Ovariohysterectomy or ovariectomy	Secondary
Perez et al (83)	*2013*	Clinical	Controlled	Castration	Primary
Perry et al (84)	*2015*	Clinical	Controlled	Distal limbs orthopaedic surgery	Secondary
Portela et al (85)	*2012*	Clinical	Observational	Orthopaedic surgery of the pelvic limb	Secondary
Re Bravo et al (86)	*2019*	Clinical	Controlled	Hemilaminectomy (single acute vertebral disc extrusion)	Primary
Read et al (87)	*2019*	Experimental	Controlled	Lateral thoracotomy	Primary

Reece et al (88)	*2012*	Clinical	Observational	Ovariohysterectomy	Secondary
Rioja et al (89)	*2012*	Clinical	Controlled	Ovariohysterectomy	Secondary
Romano et al (90)	*2016*	Clinical	Controlled	TPLO	Secondary
Sarotti et al (91)	*2015*	Clinical	Controlled	Pelvic limb orthopaedic surgery above the knee	Secondary
Sarotti et al (92)	*2019*	Clinical	Controlled	Hindlimb surgery	Secondary
Scott et al (93)	*2018*	Experimental	Controlled	Laparoscopy	Secondary
Shah et al (94)	*2018*	Clinical	Controlled	Ovariohysterectomy	Primary
Shih et al (95)	*2008*	Clinical	Controlled	Ovariohysterectomy	Primary
Shilo-Benjamini et al (96)	*2019*	Clinical	Controlled	Enucleation	Primary
Shivley et al (97)	*2018*	Clinical	Controlled	Ovariohysterectomy (suspensory ligament: sharp transection or digital strumming)	Secondary
Skelding et al (98)	*2019*	Clinical	Controlled	Various surgical procedures of the thoracic limb	Primary
Srithunyarat et al (99)	*2016*	Clinical	Observational	Ovariohysterectomy	Primary
Swallow et al (100)	*2017*	Clinical	Controlled	Ovariohysterectomy	Secondary
Tallant et al (101)	*2016*	Clinical	Controlled	Ovariohysterectomy/Ovariectomy	Primary
Tayari et al (102)	*2017*	Clinical	Controlled	TPLO	Primary
Tayari et al (103)	*2019*	Clinical	Observational	Thoracic limb orthopaedic surgery (distal to the mid-humerus)	Secondary
Travis et al (104)	*2017*	Clinical	Controlled	Midline celiotomy	Secondary
Valtolina et al (105)	*2009*	Clinical	Controlled	Exploratory laparotomy, thoracotomy, orthopaedic surgery	Primary
Vettorato et al (106)	*2010*	Clinical	Controlled	TPLO	Primary

Wagner et al (107)	*2008*	Clinical	Controlled	Castration or Ovariohysterectomy	Primary
Wagner et al (108)	*2010*	Clinical	Controlled	Forelimb amputation	Primary
Wang-Leandro et al (109)	*2019*	Experimental	Controlled	Tranresctal intraprostatic steam application	Secondary
Watanabe et al (110)	*2018*	Clinical	Controlled	Ovariohysterectomy	Primary
Weil et al (111)	*2016*	Clinical	Controlled	Soft tissue or orthopaedic surgery	Secondary
Zhang et al (112)	*2017*	Clinical	Controlled	Ovariohysterectomy	Primary
Zidan et al (113)	*2018*	Clinical	Controlled	Hemilaminectomy	Secondary
Zidan et al (114)	*2018*	Clinical	Controlled	Hemilaminectomy	Secondary

Supplementary References

1. Adami C, Casoni D, Noussitou F, Rytz U, Spadavecchia C. Addition of magnesium sulphate to ropivacaine for spinal analgesia in dogs undergoing tibial plateau levelling osteotomy. *Vet J* (2016) **209**:163–168. doi:10.1016/j.tvjl.2015.11.017

2. Adami C, Veres-Nyéki K, Spadavecchia C, Rytz U, Bergadano A. Evaluation of peri-operative epidural analgesia with ropivacaine, ropivacaine and sufentanil, and ropivacaine, sufentanil and epinephrine in isoflurane anesthetized dogs undergoing tibial plateau levelling osteotomy. *Vet J* (2012) **194**:229–234. doi:10.1016/j.tvjl.2012.04.019

3. Aengwanich W, Sakundech K, Chompoosan C, Tuchpramuk P, Boonsorn T. Physiological changes, pain stress, oxidative stress, and total antioxidant capacity before, during, and after castration in male dogs. *Journal of Veterinary Behavior* (2019) **32**:76–79. doi:10.1016/j.jveb.2019.04.004

4. Aghighi SA, Tipold A, Piechotta M, Lewczuk P, Kästner SBR. Assessment of the effects of adjunctive gabapentin on postoperative pain after intervertebral disc surgery in dogs. *Vet Anaesth Analg* (2012) **39**:636–646. doi:10.1111/j.1467-2995.2012.00769.x

5. Amengual M, Leigh H, Rioja E. Postoperative respiratory effects of intravenous fentanyl compared to intravenous methadone in dogs following spinal surgery. *Vet Anaesth Analg* (2017) **44**:1042–1048. doi:10.1016/j.vaa.2016.11.010

6. Andreoni V, Hughes JML. Propofol and fentanyl infusions in dogs of various breeds undergoing surgery. *Veterinary Anaesthesia and Analgesia* (2009) **36**:523–531. doi:10.1111/j.1467-2995.2009.00490.x

7. Aprea F, Cherubini GB, Palus V, Vettorato E, Corletto F. Effect of extradurally administered morphine on postoperative analgesia in dogs undergoing surgery for thoracolumbar intervertebral disk extrusion. *Scientific Reports* (2012) **241**:6.

8. Barker J, Clark-Price S. Evaluation of Topical Epidural Analgesia Delivered in Gelfoam for Postoperative Hemilaminectomy Pain Control. *Veterinary Surgery* (2013)7.

9. Barnes K, Faludi A, Takawira C, Aulakh K. Extracorporeal Shock Wave Therapy Improves Short Term Limb use after Canine Tibial Plateau Leveling Osteotomy. (2019)10.

10. Bartel AK, Campoy L, Martin-Flores M, Gleed RD, Walker KJ, Scanapico CE, Reichard AB. Comparison of bupivacaine and dexmedetomidine femoral and sciatic nerve blocks with bupivacaine and buprenorphine epidural injection for stifle arthroplasty in dogs. *Veterinary Anaesthesia and Analgesia* (2016) **43**:435–443. doi:10.1111/vaa.12318

11. Bellei E, Roncada P, Pisoni L, Joechler M, Zaghini A. The use of fentanylpatch in dogs undergoing spinal surgery: plasma concentration and analgesic efficacy. (2011)5.

12. Bendinelli C, Properzi R, Boschi P, Bresciani C, Rocca E, Sabbioni A, Leonardi F. Meloxicam vs robenacoxib for postoperative pain management in dogs undergoing combined laparoscopic ovariectomy and laparoscopic-assisted gastropexy. (2018)6.

13. Benitez ME, Roush JK, McMurphy R, KuKanich B, Legallet C. Clinical efficacy of hydrocodone-acetaminophen and tramadol for control of postoperative pain in dogs following tibial plateau leveling osteotomy. *American Journal of Veterinary Research* (2015) **76**:755–762. doi:10.2460/ajvr.76.9.755

14. Benitez ME, Roush JK, KuKanich B, McMurphy R. Pharmacokinetics of hydrocodone and tramadol administered for control of postoperative pain in dogs following tibial plateau leveling osteotomy. (2015) **76**:8.

15. Bienhoff SE, Smith ES, Roycroft LM, Roberts ES, Baker LD. Efficacy and Safety of Deracoxib for the Control of Postoperative Pain and Inflammation Associated with Dental Surgery in Dogs. *ISRN Veterinary Science* (2011)8.

16. Bienhoff SE, Smith ES, Roycroft LM, Roberts ES. Efficacy and Safety of Deracoxib for Control of Postoperative Pain and Inflammation Associated with Soft Tissue Surgery in Dogs. *Veterinary Surgery* (2012)9.

17. Bustamante R, Daza M, Canfran S, Garcia P, Suarez M, Trobo I, Gomez de Segura I. Comparison of the postoperative analgesic effects of cimicoxib, buprenorphine and their combination in healthy dogs undergoing ovariohysterectomy. (2018)12.

18. Caniglia AM, Driessen B, Puerto DA, Bretz B, Boston RC, Larenza MP. Intraoperative antinociception and postoperative analgesia following epidural anesthesia versus femoral and sciatic nerve blockade in dogs undergoing stifle joint surgery. *Journal of the American Veterinary Medical Association* (2012) **241**:1605–1612. doi:10.2460/javma.241.12.1605

19. Cerasoli I, Tutunaru A, Cenani A, Ramirez J, Detilleux J, Belligand M, Sendersen C. Comparison of clinical effects of epidural levobupivacaine morphine versus bupivacaine morphine in dogs undergoing elective pelvic limb surgery. (2017)9.

20. Chiavaccini L, Claude AK, Meyer RE. Comparison of Morphine, Morphine-Lidocaine, and Morphine-Lidocaine-Ketamine Infusions in Dogs Using an Incision-Induced Pain Model. *Journal of the American Animal Hospital Association* (2017) **53**:65–72. doi:10.5326/JAAHA-MS-6442

21. Dancker C, MacFarlane PD, Love EJ. The effect of neuraxial morphine on postoperative pain in dogs after extrahepatic portosystemic shunt attenuation. *Veterinary Anaesthesia and Analgesia* (2020) **47**:111–118. doi:10.1016/j.vaa.2019.06.011

22. Davila D, Keeshen TP, Evans RB, Conzemius MG. Comparison of the analgesic efficacy of perioperative firocoxib and tramadol administration in dogs undergoing tibial plateau leveling osteotomy. *Journal of the American Veterinary Medical Association* (2013) **243**:225–231. doi:10.2460/javma.243.2.225

23. Fitzpatrick CL, Weir HL, Monnet E. Effects of infiltration of the incision site with bupivacaine on postoperative pain and incisional healing in dogs undergoing ovariohysterectomy. *Journal of the American Veterinary Medical Association* (2010) **237**:395–401. doi:10.2460/javma.237.4.395

24. Fizzano KM, Claude AK, Kuo L-H, Eells JB, Hinz SB, Thames BE, Ross MK, Linford RL, Wills RW, Olivier AK, et al. Evaluation of a modified infraorbital approach for a maxillary nerve block for rhinoscopy with nasal biopsy of dogs. (2017) **78**:11.

25. Fransson BA, Perez TE, Flores K, Gay JM, Acvpm D. Cardiorespiratory Changes and Pain Response of Lift Laparoscopy Compared to Capnoperitoneum Laparoscopy in Dogs. *Veterinary Surgery* (2015)9.

26. Friton G, Thompson C, Karadzovska D, King S, King JN. Efficacy and safety of oral robenacoxib (tablet) for the treatment of pain associated with soft tissue surgery in client-owned dogs. (2017)12.

27. Friton G, Thompson C, Karadzovska D, King S, King JN. Efficacy and Safety of Injectable Robenacoxib for the Treatment of Pain Associated With Soft Tissue Surgery in Dogs. (2017)10.

28. Giudice E, Barillaro G, Crinò C, Alaimo A, Macrì F, Di Pietro S. Postoperative pain in dogs undergoing hemilaminectomy: Comparison of the analgesic activity of buprenorphine and tramadol. *Journal of Veterinary Behavior* (2017) **19**:45–49. doi:10.1016/j.jveb.2017.02.003

29. Goudie-DeAngelis EM, Woodhouse KJ. Evaluation of Analgesic Efficacy and Associated Plasma Concentration of Tramadol and O-desmethyltramadol Following Oral Administration Post Ovariohysterectomy. (2016) **14**:9.

30. Groppetti D, Di Cesare F, Pecile A, Cagnardi P, Merlanti R, D'Urso E, Gioeni D, Boracchi P, Ravasio G. Maternal and neonatal wellbeing during elective C-section induced with a combination of propofol and dexmedetomidine: How effective is the placental barrier in dogs? (2019)9.

31. Gruet P, Seewald W, King JN. Evaluation of subcutaneous and oral administration of robenacoxib and meloxicam for the treatment of acute pain and inflammation associated with orthopedic surgery in dogs. (2011) **72**:10.

32. Gruet P, Seewald W, King JN. Robenacoxib versus meloxicam for the management of pain and inflammation associated with soft tissue surgery in dogs: a randomized, non-inferiority clinical trial. (2013)12.

33. Guerrero KSK, Schwarz A, Wuhrmann R, Feldmann S, Hartnack S, Bettschart-Wolfensberger R. Comparison of a new metamizole formulation and carprofen for extended post-operative analgesia in dogs undergoing ovariohysterectomy. *The Veterinary Journal* (2015)6.

34. Guerrero KSK, Campagna I, Bruhl-Day R, Hegamin-Younger C, Guerrero T. Intraperitoneal bupivacaine with or without incisional bupivacaine for postoperative analgesia in dogs undergoing ovariohysterectomy. (2016)8.

35. Guillot M, Rialland P, Nadeau M-È, del Castillo JRE, Gauvin D, Troncy E. Pain Induced by a Minor Medical Procedure (Bone Marrow Aspiration) in Dogs: Comparison of Pain Scales in a Pilot Study. *Journal of Veterinary Internal Medicine* (2011) **25**:1050–1056. doi:10.1111/j.1939-1676.2011.00786.x

36. Alves IPG, Nicácio GM, Diniz MS, Rocha TLA, Prada G, Cassu RN. Analgesic comparison of systemic lidocaine, morphine or lidocaine plus morphine infusion in dogs undergoing fracture repair. (2014)7.

37. Gurney MA, Rysnik M, Comerford EJ, Cripps PJ, Iff I. Intra-articular morphine, bupivacaine or no treatment for postoperative analgesia following unilateral elbow joint arthroscopy. *Journal of Small Animal Practice* (2012) **53**:6.

38. Gutiérrez-Bautista ÁJ, Morgaz J, Granados M del M, Gómez-Villamandos RJ, Dominguez JM, Fernandez-Sarmiento JA, Aguilar-García D, Navarrete-Calvo R. Evaluation and comparison of postoperative analgesic effects of dexketoprofen and methadone in dogs. *Veterinary Anaesthesia and Analgesia* (2018) **45**:820–830. doi:10.1016/j.vaa.2018.06.016

39. Gutierrez-Blanco E, Victoria-Mora JM, Ibancovichi-Camarillo JA, Sauri-Arceo CH, Bolio-González ME, Acevedo-Arcique CM, Marin-Cano G, Steagall PV. Postoperative analgesic effects of either a constant rate infusion of fentanyl, lidocaine, ketamine,

dexmedetomidine, or the combination lidocaine-ketamine-dexmedetomidine after ovariohysterectomy in dogs. *Veterinary Anaesthesia and Analgesia* (2015) **42**:309–318. doi:10.1111/vaa.12215

40. Hamilton KH, Henderson ER, Toscano M, Chanoit GP. Comparison of postoperative complications in healthy dogs undergoing open and closed orchidectomy. *J Small Anim Pract* (2014) **55**:521–526. doi:10.1111/jsap.12266

41. Heffernan AE, Katz EM, Sun Y, Rendahl AK, Conzemius MG. Once daily oral extended-release hydrocodone as analgesia following tibial plateau leveling osteotomy in dogs. *Veterinary Surgery* (2018) **47**:516–523. doi:10.1111/vsu.12792

42. Hettlich BF, Cook L, London C, Fosgate GT. Comparison of harmonic blade versus traditional approach in canine patients undergoing spinal decompressive surgery for naturally occurring thoracolumbar disk extrusion. *PLoS ONE* (2017) **12**:e0172822. doi:10.1371/journal.pone.0172822

43. Hu XY, Luan L, Guan W, Shi J, Zhao YB, Fan HG. Tolfenamic acid and meloxicam both provide an adequate degree of postoperative analgesia in dogs undergoing ovariohysterectomy. *Veterinarni Medicina* (2017)9.

44. Hunt JR, Attenburrow PM, Slingsby LS, Murrell JC. Comparison of premedication with buprenorphine or methadone with meloxicam for postoperative analgesia in dogs undergoing orthopaedic surgery. *J Small Anim Pract* (2013) **54**:418–424. doi:10.1111/jsap.12103

45. Hunt JR, Grint NJ, Taylor PM, Murrell JC. Sedative and analgesic effects of buprenorphine, combined with either acepromazine or dexmedetomidine, for premedication prior to elective surgery in cats and dogs. *Veterinary Anaesthesia and Analgesia* (2013) **40**:297–307. doi:10.1111/vaa.12003

46. Hunt JR, Slingsby LS, Murrell JC. The effects of an intravenous bolus of dexmedetomidine following extubation in a mixed population of dogs undergoing general anaesthesia and surgery. *The Veterinary Journal* (2014) **200**:133–139. doi:10.1016/j.tvjl.2014.01.015

47. Huuskonen V, Hughes JL, Estaca Bañon E, West E. Intratesticular lidocaine reduces the response to surgical castration in dogs. *Veterinary Anaesthesia and Analgesia* (2013) **40**:74–82. doi:10.1111/j.1467-2995.2012.00775.x

48. Kaka U, Rahman N-A, Abubakar AA, Goh YM, Fakurazi S, Omar MA, Chen HC. Pre-emptive multimodal analgesia with tramadol and ketamine-lidocaine infusion for suppression of central sensitization in a dog model of ovariohysterectomy. *JPR* (2018) **Volume 11**:743–752. doi:10.2147/JPR.S152475

49. Karrasch NM, Lerche P, Aarnes TK, Gardner HL, London CA. The effects of preoperative oral administration of carprofen or tramadol on postoperative analgesia in dogs undergoing cutaneous tumor removal. (2015) **56**:6.

50. Kibar M, Tuna B, Kisadere I, Güzelbektes H. Comparison of Instilled Lidocaine and Procaine Effects on Pain Relief in Dogs Undergoing Elective Ovariohysterectomy. *Israel Journal of Veterinary Medicine* (2019) **74**: Available at: https://www.ivis.org/library/israel-journal-of-veterinary-medicine/israel-journal-of-veterinary-medicine-vol-743-sep-7 [Accessed January 23, 2021]

51. Kim JH, Seok SH, Park TY, Kim HJ, Lee SW, Lee HC, Yeon SC. Analgesic effect of intra-articular ropivacaine injection after arthroscopic surgery on the shoulder joint in dogs. *Veterinarni Medicina* (2018) **63**:513–521. doi:10.17221/37/2017-VETMED

52. Kim YK, Lee S, Suh E, Lee L, Lee H, Lee H, Yeon SC. Sprayed intraperitoneal bupivacaine reduces early postoperative pain behavior and biochemical stress response after laparoscopic ovariohysterectomy in dogs. *The Veterinary Journal* (2012)5.

53. Kondo Y, Takashima K, Matsumoto S, Shiba M, Otsuki T, Kinoshita G, Rosentel J, Gross SJ, Fleishman C, Yamane Y. Efficacy and Safety of Firocoxib for the Treatment of Pain Associated with Soft Tissue Surgery in Dogs under Field Conditions in Japan. (2012)7.

54. Kongara K, Chambers J, Johnson C. Effects of tramadol, morphine or their combination in dogs undergoing ovariohysterectomy on peri-operative electroencephalographic responses and post-operative pain. *New Zealand Veterinary Journal* (2012) **60**:129–135. doi:10.1080/00480169.2011.641156

55. Kongara K, Chambers J, Johnson C, Dukkipati V. Effects of tramadol or morphine in dogs undergoing castration on intra-operative electroencephalogram responses and post-operative pain. *New Zealand Veterinary Journal* (2013) **61**:349–353. doi:10.1080/00480169.2013.780280

56. Kropf J, Hughes JML. Effects of midazolam on cardiovascular responses and isoflurane requirement during elective ovariohysterectomy in dogs. *Ir Vet J* (2018) **71**:26. doi:10.1186/s13620-018-0136-y

57. Kropf J, Hughes JML. Effect of midazolam on the quality and duration of anaesthetic recovery in healthy dogs undergoing elective ovariohysterectomy or castration. (2019)10.

58. Kushnir Y, Toledano N, Cohen L, Bdolah-Abram T, Shilo-Benjamini Y. Intratesticular and incisional line infiltration with ropivacaine for castration in medetomidine-butorphanol-midazolam sedated dogs. (2017)10.

59. Lambertini C, Kluge K, Lanza-Perea M, Bruhl-Day R, Guerrero KSK. Comparison of intraperitoneal ropivacaine and bupivacaine for postoperative analgesia in dogs undergoing ovariohysterectomy. (2018)6.

60. Lardone E, Peirone B, Adami C. Combination of magnesium sulphate and ropivacaine epidural analgesia for hip arthroplasty in dogs. (2017)9.

61. Lascelles BDX, Rausch-Derra LC, Wofford JA, Huebner M. Pilot, randomized, placebo-controlled clinical field study to evaluate the effectiveness of bupivacaine liposome injectable suspension for the provision of post-surgical analgesia in dogs undergoing stifle surgery. *BMC Vet Res* (2016) **12**:168. doi:10.1186/s12917-016-0798-1

62. Lewis KA, Bednarski RM, Aarnes TK, Dyce J, Hubbell JAE. Postoperative comparison of four perioperative analgesia protocols in dogs undergoing stifle joint surgery. *Journal of the American Veterinary Medical Association* (2014) **244**:1041–1046. doi:10.2460/javma.244.9.1041

63. Li L, Dong J, Fen X, Li B, Chen Y, Sha J, Fan H. Effects of dexmedetomidine on plasma glucose, cortisol and adrenocorticotropic hormone concentrations of canine undergoing ovariohysterectomy. *Thai J Vet Med* (2017)6.

64. Linton DD, Wilson MG, Newbound GC, Freise KJ, Clark TP. The effectiveness of a long-acting transdermal fentanyl solution compared to buprenorphine for the control of postoperative pain in dogs in a randomized, multicentered clinical study. *Journal of Veterinary Pharmacology and Therapeutics* (2012) **35**:53–64. doi:10.1111/j.1365-2885.2012.01408.x

65. Little D, Johnson S, Hash J, Olson SA, Estes BT, Moutos FT, Lascelles BDX, Guilak F. Functional outcome measures in a surgical model of hip osteoarthritis in dogs. *J EXP ORTOP* (2016) **3**:17. doi:10.1186/s40634-016-0053-5

66. Luna SPL, Martino I, Lorena S, Capua M, Lima A, Santos B, Brondani J, Vesce G. Acupuncture and pharmacopuncture are as effective as morphine or carprofen for postoperative analgesia in bitches undergoing ovariohysterectomy. (2015)7.

67. Lush J, Ijichi C. A preliminary investigation into personality and pain in dogs. (2018)7.

68. Martinez SA, Wilson MG, Linton DD, Newbound GC, Freise KJ, Lin T -L., Clark TP. The safety and effectiveness of a long-acting transdermal fentanyl solution compared with oxymorphone for the control of postoperative pain in dogs: a randomized, multicentered clinical study. *J vet Pharmacol Therap* (2014) **37**:394–405. doi:10.1111/jvp.12096

69. Martinez-Taboada F, Redondo JI. Comparison of the hanging-drop technique and running-drip method for identifying the epidural space in dogs. *Veterinary Anaesthesia and Analgesia* (2017) **44**:329–336. doi:10.1016/j.vaa.2016.03.002

70. McCally RE, Bukoski A, Branson KR, Fox DB, Cook JL. Comparison of Short-Term Postoperative Analgesia by Epidural, Femoral Nerve Block, or Combination Femoral and Sciatic Nerve Block in Dogs Undergoing Tibial Plateau Leveling Osteotomy: Comparison of Epidural or Peripheral Nerve Blocks After TPLO. *Veterinary Surgery* (2015) **44**:983–987. doi:10.1111/vsu.12406

71. McKune CM, Pascoe PJ, Lascelles BDX, Kass PH. The challenge of evaluating pain and a pre-incisional local anesthetic block. *PeerJ* (2014) **2**:e341. doi:10.7717/peerj.341

72. McMillan MW, Seymour CJ, Brearley JC. Effect of intratesticular lidocaine on isoflurane requirements in dogs undergoing routine castration. *Journal of Small Animal Practice* (2012) **53**:5.

73. Meakin LB, Murrell JC, Doran ICP, Knowles TG, Tivers MS, Chanoit GPA. Electrosurgery reduces blood loss and immediate postoperative inflammation compared to cold instruments for midline celiotomy in dogs: A randomized controlled trial. (2016)5.

74. Merema DK, Schoenrock EK, Boedec KL, McMichael MA. Effects of a transdermal lidocaine patch on indicators of postoperative pain in dogs undergoing midline ovariohysterectomy. (2017) **250**:8.

75. Meunier NV, Panti A, Mazeri S, Fernandes KA, Handel IG. Randomised trial of perioperative tramadol for canine sterilisation pain management. (2019)8.

76. Morgaz J, Navarrete R, Muñoz-Rascón P, Domínguez JM, Fernández-Sarmiento JA, Gómez-Villamandos RJ, Granados MM. Postoperative analgesic effects of dexketoprofen, buprenorphine and tramadol in dogs undergoing ovariohysterectomy. *Research in Veterinary Science* (2013) **95**:278–282. doi:10.1016/j.rvsc.2013.03.003

77. Morgaz J, Muñoz-Rascón P, Serrano-Rodriguez J, Navarrete R, Dominguez JM, Fernandez-Sarmiento JA, Gomez-Villamandos R, Serrano J, Granados MM. Effectiveness of pre-peritoneal continuous wound infusion with lidocaine for pain control following ovariohysterectomy in dogs. *The Veterinary Journal* (2014)5.

78. Nour E, Othman M, Karrouf G, Zaghloul A. Glasgow Composite Measure Pain Scale score and comaprison between several adjuvants in association with bupivacaine. *Life Science Journal* (2013) **10**: Available at: https://www.researchgate.net/publication/256399795_Glasgow_Composite_Measure_Pain_Scale_score_and_comaprison_between_several_adjuvants_in_association_with_bupivacaine [Accessed January 23, 2021]

79. Palomba N, Vettorato E, De Gennaro C, Corletto F. Peripheral nerve block versus systemic analgesia in dogs undergoing tibial plateau levelling osteotomy: Analgesic efficacy and pharmacoeconomics comparison. (2019)10.

80. Pascal M, Burac M, Diaconescu A, Togoe D, Vitalaru A, Bîrtoiu A. Comparison of tramadol and robenacoxib postoperative analgesic efficacy in dogs. *Scientific Works Series C Veterinary Medicine* (2013) **LIX**:72–75.

81. Pascal M, Allison A, Kaartinen J. Opioid-sparing effect of a medetomidine constant rate infusion during thoraco-lumbar hemilaminectomy in dogs administered a ketamine infusion. *Veterinary Anaesthesia and Analgesia* (2020) **47**:61–69. doi:10.1016/j.vaa.2019.06.012

82. Peeters ME, Kirpensteijn J. Comparison of surgical variables and short-term postoperative complications in healthy dogs undergoing ovariohysterectomy or ovariectomy. *Journal of the American Veterinary Medical Association* (2011) **238**:189–194. doi:10.2460/javma.238.2.189

83. Perez TE, Grubb TL, Greene SA, Meyer S, Valdez N, Bingman J, Farnsworth R. Effects of intratesticular injection of bupivacaine and epidural administration of morphine in dogs undergoing castration. *Scientific Reports* (2013) **242**:12.

84. Perry K, Rutherford L, Sajik D, Bruce M. A preliminary study of the effect of closed incision management with negative pressure wound therapy over high-risk incisions. (2015)12.

85. Portela D, Otero P, Briganti A, Romano M, Corletto F, Breghi G. Femoral nerve block: a novel psoas compartment lateral pre-iliac approach in dogs. (2012)11.

86. Re Bravo V, Aprea F, Bhalla RJ, De Gennaro C, Cherubini GB, Corletto F, Vettorato E. Effect of 5% transdermal lidocaine patches on postoperative analgesia in dogs undergoing hemilaminectomy. *J Small Anim Pract* (2019) **60**:161–166. doi:10.1111/jsap.12925

87. Read K, Khatun M, Murphy H. Comparison of transdermal fentanyl and oral tramadol for lateral thoracotomy in dogs: cardiovascular and behavioural data. *Veterinary Anaesthesia and Analgesia* (2019) **46**:116–125. doi:10.1016/j.vaa.2018.09.046

88. Reece JF, Nimesh MK, Wyllie RE, Jones AK, Dennison AW. Description and evaluation of a right flank, mini-laparotomy approach to canine ovariohysterectomy. *Veterinary Record* (2012) **171**:248–248. doi:10.1136/vr.100907

89. Rioja E, Dzikiti B, Fosgate G, Goddaard A, Stegmann F, Schoeman J. Effects of a constant rate infusion of magnesium sulphate in healthy dogs anaesthetized with isoflurane and undergoing ovariohysterectomy. (2012)12.

90. Romano M, Portela DA, Breghi G, Otero PE. Stress-related biomarkers in dogs administered regional anaesthesia or fentanyl for analgesia during stifle surgery. *Veterinary Anaesthesia and Analgesia* (2016) **43**:44–54. doi:10.1111/vaa.12275

91. Sarotti D, Rabozzi R, Franci P. Comparison of epidural versus intrathecal anaesthesia in dogs undergoing pelvic limb orthopaedic surgery. *Veterinary Anaesthesia and Analgesia* (2015) **42**:405–413. doi:10.1111/vaa.12229

92. Sarotti D, Rabozzi R, Franci P. Effects of intravenous dexmedetomidine infusion on local anaesthetic block: A spinal anaesthesia clinical model in dogs undergoing hind limb surgery. *Research in Veterinary Science* (2019) **124**:93–98. doi:10.1016/j.rvsc.2019.03.001

93. Scott JE, Singh A, Valverde A, Blois SL, Foster RA, Kilkenny JJ, Linden A zur. Effect of pneumoperitoneum with warmed humidified or standard-temperature carbon dioxide during laparoscopy on core body temperature, cardiorespiratory and thromboelastography variables, systemic inflammation, peritoneal response, and signs of postoperative pain in healthy mature dogs. *American Journal of Veterinary Research* (2018) **79**:1321–1334. doi:10.2460/ajvr.79.12.1321

94. Shah MD, Yates D, Hunt J, Murrell JC. A comparison between methadone and buprenorphine for perioperative analgesia in dogs undergoing ovariohysterectomy: A comparison of methadone and buprenorphine. *J Small Anim Pract* (2018) **59**:539–546. doi:10.1111/jsap.12859

95. Shih AC, Robertson S, Isaza N, Pablo L, Davies W. Comparison between analgesic effects of buprenorphine, carprofen, and buprenorphine with carprofen for canine ovariohysterectomy. *Veterinary Anaesthesia and Analgesia* (2008) **35**:69–79. doi:10.1111/j.1467-2995.2007.00352.x

96. Shilo-Benjamini Y, Slav SA, Kahane N, Kushnir Y, Sarfaty H, Ofri R. Analgesic effects of intraorbital insertion of an absorbable gelatin hemostatic sponge soaked with 1% ropivacaine solution following enucleation in dogs. *Journal of the American Veterinary Medical Association* (2019) **255**:1255–1262. doi:10.2460/javma.255.11.1255

97. Shivley J, Richardson JM, Woodruff KA, Brookshire W, Meyer R, Smith D. Sharp Transection of the Suspensory Ligament as an Alternative to Digital Strumming during Canine Ovariohysterectomy. (2018)6.

98. Skelding A, Valverde A, Aguilera R, Moens NM, Sinclair M, Thomason JJ. Comparison of 3 blind brachial plexus block techniques during maintenance of anesthesia and postoperative pain scores in dogs undergoing surgical procedures of the thoracic limb. (2019)9.

99. Srithunyarat T, Höglund OV, Hagman R, Olsson U, Stridsberg M, Lagerstedt A-S, Pettersson A. Catestatin, vasostatin, cortisol, temperature, heart rate, respiratory rate, scores of the short form of the Glasgow composite measure pain scale and visual analog scale for stress and pain behavior in dogs before and after ovariohysterectomy. *BMC Res Notes* (2016) **9**:381. doi:10.1186/s13104-016-2193-1

100. Swallow A, Rioja E, Elmer T, Dugdale A. The effect of maropitant on intraoperative isoflurane requirements and postoperative nausea and vomiting in dogs: a randomized clinical trial. *Veterinary Anaesthesia and Analgesia* (2017) **44**:785–793. doi:10.1016/j.vaa.2016.10.006

101. Tallant A, Ambros B, Freire C, Sakals S. Comparison of intraoperative and postoperative pain during canine ovariohysterectomy and ovariectomy. (2016) **57**:6.

102. Tayari H, Tazioli G, Breghi G, Briganti A. Ultrasound-guided femoral and obturator nerves block in the psoas compartment in dogs: anatomical and randomized clinical study. (2017)11.

103. Tayari H, Otero P, Rossetti A, Breghi G, Briganti A. Proximal RUMM block in dogs: preliminary results of cadaveric and clinical studies. *Veterinary Anaesthesia and Analgesia* (2019) **46**:384–394. doi:10.1016/j.vaa.2018.11.009

104. Travis BM, Hayes GM, Vissio K, Harvey HJ, Flanders JA, Sumner JP. A quilting subcutaneous suture pattern to reduce seroma formation and pain 24 hours after midline celiotomy in dogs: A randomized controlled trial. *Veterinary Surgery* (2018) **47**:204–211. doi:10.1111/vsu.12754

105. Valtolina C, Robben JH, Uilenreef J, Murrell JC, Aspegrén J, McKusick BC, Hellebrekers LJ. Clinical evaluation of the efficacy and safety of a constant rate infusion of dexmedetomidine for postoperative pain management in dogs. *Veterinary Anaesthesia and Analgesia* (2009) **36**:369–383. doi:10.1111/j.1467-2995.2009.00461.x

106. Vettorato E, Zonca A, Isola M, Villa R, Gallo M, Ravasio G, Beccaglia M, Montesissa C, Cagnardi P. Pharmacokinetics and efficacy of intravenous and extradural tramadol in dogs. *The Veterinary Journal* (2010) **183**:310–315. doi:10.1016/j.tvjl.2008.11.002

107. Wagner AE, Worland GA, Glawe JC, Hellyer PW. Multicenter, randomized controlled trial of pain-related behaviors following routine neutering in dogs. *Journal of the American Veterinary Medical Association* (2008) **233**:109–115. doi:10.2460/javma.233.1.109

108. Wagner AE, Mich PM, Uhrig SR, Hellyer PW. Clinical evaluation of perioperative administration of gabapentin as an adjunct for postoperative analgesia in dogs undergoing amputation of a forelimb. *Journal of the American Veterinary Medical Association* (2010) **236**:751–756. doi:10.2460/javma.236.7.751

109. Wang-Leandro A, Willmitzer F, Karol A, Porcellini B, Kronen P, Hiltbrand EM, Rüfenacht D, Kircher PR, Richter H. MRI-guided, transrectal, intraprostatic steam application as potential focal therapeutic modality for prostatic diseases in a large animal translational model: A feasibility follow-up study. *PLoS ONE* (2019) **14**:e0226764. doi:10.1371/journal.pone.0226764

110. Watanabe R, Monteiro BP, Evangelista MC, Castonguay A, Edge D, Steagall PV. The analgesic effects of buprenorphine (Vetergesic or Simbadol) in combination with carprofen in dogs undergoing ovariohysterectomy: a randomized, blinded, clinical trial. *BMC Vet Res* (2018) **14**:304. doi:10.1186/s12917-018-1628-4

111. Weil C, Tünsmeyer J, Tipold A, Hoppe S, Beyerbach M, Pankow W-R, Kästner SB. Effects of concurrent perioperative use of marbofloxacin and cimicoxib or carprofen in dogs: Marbofloxacin and cimicoxib or carprofen in dogs. *J Small Anim Pract* (2016) **57**:311–317. doi:10.1111/jsap.12464

112. Zhang S, Li J, Luan L, Guan W, Hu X, Fan H. Comparison of the effects of nefopam and tramadol on postoperative analgesia in dogs undergoing ovariohysterectomy. *Veterinarni Medicina* (2017) **62**:131–137. doi:10.17221/53/2016-VETMED

113. Zidan N, Fenn J, Griffith E, Early PJ, Mariani CL, Muñana KR, Guevar J, Olby NJ. The Effect of Electromagnetic Fields on Post-Operative Pain and Locomotor Recovery in Dogs with Acute, Severe Thoracolumbar Intervertebral Disc Extrusion: A Randomized Placebo-Controlled, Prospective Clinical Trial. *Journal of Neurotrauma* (2018) **35**:1726–1736. doi:10.1089/neu.2017.5485

114. Zidan N, Sims C, Fenn J, Williams K, Griffith E, Early PJ, Mariani CL, Munana KR, Guevar J, Olby NJ. A randomized, blinded, prospective clinical trial of postoperative rehabilitation in dogs after surgical decompression of acute thoracolumbar intervertebral disc herniation. *J Vet Intern Med* (2018) **32**:1133–1144. doi:10.1111/jvim.15086
