# Supplementary material for: The Short Form of the Glasgow Composite Measure Pain Scale in Post-operative Analgesia Studies in Dogs: A Scoping Review
Source: Front Vet Sci. 2021 Sep 30;8:751949. doi: 10.3389/fvets.2021.751949 (PMC8515184; doi:10.3389/fvets.2021.751949)
Supplement: Supplementary file 2 [file Data_Sheet_2.pdf]

Supplementary material 2. Search strategy

Search strategy	A systematic search of four bibliographic databases (PubMed, CAB abstracts, Web of Science and Google Scholar) was conducted for papers published between 2007 and 2019 (inclusive) using either Safari or Google Chrome as web browsers. Searches were carried out on each database using a combination of the following key words (and derivatives): dogs (dog *OR* dogs) *AND* the Glasgow Composite Measure Pain Scale - short form (GCMPS-SF *OR* GCMPS *OR* CMPS *OR* CMPS-SF *OR* Glasgow Composite Measure Pain Scale *OR* GCMPS short form *OR* CMPS short form *OR* GCPS) *AND* postoperative (post operative *OR* post-operative *OR* postoperative) *AND* pain.<br>We first conducted restricted searches of titles and abstracts based on the terms 'dog' *AND* 'CMPS-SF' *AND* 'postoperative' AND 'pain', subsequently broadening our searches using the terms 'CMPS-SF' *AND* "dog' and their derivatives. However, neither of these search results contained several papers that the authors knew of that would have fully satisfied the inclusion criteria. Therefore, we adopted an additional broader search strategy using the terms 'postoperative' *AND* 'pain' *AND* 'dog' and their derivatives. As an example the detailed Pubmed search strategy is given below. Additional studies were identified by browsing the reference list of the included papers and by using the citing articles search feature in Google Scholar and Web of Science to identify any articles citing the original paper describing the development of the CMPS-SF (10).
Inclusion criteria	Each publication was initially assessed against the inclusion and exclusion criteria based on the title, abstract and further reading if necessary. Publications were included if they met the following criteria: (i) use of the Glasgow CMPS-SF to assess pain;<br>(ii) investigating acute postoperative pain; (iii) prospective design; (iv) use of the English language; (v) published in a peer-reviewed journal; (vi) conducted in dogs, and (vii) available in full to the authors.

Pubmed search strategy. Keyword searches in the title and abstract of articles are marked with the syntax [tiab]. Results from searches #8, #9 and #10 were assessed further against inclusion criteria.

Search #	Search strategy
#1	"Dog" [tiab] OR "Dogs" [tiab]
#2	"Glasgow Composite Measure Pain Scale – Short Form" [tiab] OR "GCMPS-SF" [tiab] OR "GCMPS" [tiab] OR "GCMPS short form" [tiab] OR "CMPS-

	SF” [tiab] OR “CMPS” [tiab] OR “CMPS short form” [tiab] OR “Glasgow Composite Measure Pain Scale” [tiab] OR “GCPS” [tiab]
#3	“Postoperative” [tiab] OR “Post-operative” [tiab] OR “Post operative” [tiab]
#4	“Pain” [tiab]
#5	#1 AND #2 AND #3 AND #4
#6	#1 AND #2
#7	#1 AND #3 AND #4
#8	#5 Filters: English; 2007:2019
#9	#6 Filters: English; 2007:2019
#10	#7 Filters: English; 2007:2019
